# Supplementary material for: CM1-driven assembly and activation of yeast γ-tubulin small complex underlies microtubule nucleation
Source: eLife. 2021 May 5;10:e65168. doi: 10.7554/eLife.65168 (PMC8099430; doi:10.7554/eLife.65168)
Supplement: Supplementary file 2. [file elife-65168-supp2.docx]

| **Name** | **γTuSC monomer** | **γTuSC dimer** | **γTuRC^ss^** | **γTuRC^WT^ open** | **γTuRC^WT^ closed** |
| --- | --- | --- | --- | --- | --- |
| PDB ID | 7M2Z |  | 7M2W | 7M2X | 7M2Y |
| EMDB ID | EMD-23638 | EMD-23639 | EMD-23635 | EMD-23636 | EMD-23637 |
|  | **Data collection/processing** | | | | |
| Microscope | Polara | Polara | Krios | Krios | Krios |
| Camera | K2 | K2 | K2 | K2 | K2 |
| Magnification | 39891 | 39891 | 47214 | 47214 | 47214 |
| Voltage (kV) | 300 | 300 | 300 | 300 | 300 |
| Total electron dose (e^-^/Å^2^) | 81 | 81 | 72 | 80 | 80 |
| Defocus range (µm) | 0.4-5.2 | 0.4-5.2 | 0.5-2.5 | 0.6-3.0 | 0.6-3.0 |
| Processing pixel size (Å) | 0.6267 | 0.6267 | 1.059 | 1.4183 | 1.4183 |
| Pixel size (Å)  Physical/(Super-resolution) | 1.2534/  (0.6267) | 1.2534/  (0.6267) | 1.059/  (0.5295) | 1.059/  (0.5295) | 1.059/  (0.5295) |
| Micrographs | 7381 | 7381 | 3024 | 2204 | 2204 |
| Final particles | 398841 | 506014 | 148911 | 36251 | 20420 |
| Symmetry | C1 | C1 | C1 | C1 | C1 |
| High resolution limit used in refinement | 6.0 | 8.0 | 3.7 | 4.5 | 4.5 |
| Resolution Å (FSC 0.143) | 3.7 | 4.5 | 3.0 | 3.6 | 4.0 |
|  | **Model Composition** | | | | |
| Non-hydrogen atoms | 18332 |  | 39371 | 18706 | 18563 |
| Protein residues | 2253 |  | 4827 | 2397 | 2278 |
| Ligands | 2 |  | 4 | 2 | 2 |
|  | **Model Refinement** | | | | |
| Map-model FSC (0.5) | 4.2 |  | 3.4 | 4.2 | 4.5 |
| RMSD bond lengths (Å) | 0.007 |  | 0.005 | 0.022 | 0.022 |
| RMSD bond angles (degrees) | 1.141 |  | 1.011 | 1.760 | 1.793 |
|  | **Validation** | | | | |
| MolProbity score | 2.21 |  | 1.91 | 1.09 | 1.25 |
| Clash score | 17.15 |  | 12.2 | 2.29 | 3.66 |
| C-beta deviations (%) | 0 |  | 0.02 | 0 | 0 |
| Rotamer outliers (%) | 0 |  | 0.23 | 0.14 | 0.1 |
| Ramachandran favored (%) | 92.24 |  | 95.5 | 97.62 | 97.56 |
| Ramachandran disallowed (%) | 0.04 |  | 0.02 | 0.35 | 0.27 |

**Supplementary File 2. CryoEM data collection, processing, and modeling statistics.**
