# Supplementary material for: CM1-driven assembly and activation of yeast γ-tubulin small complex underlies microtubule nucleation
Source: eLife. 2021 May 5;10:e65168. doi: 10.7554/eLife.65168 (PMC8099430; doi:10.7554/eLife.65168)
Supplement: Transparent reporting form [file elife-65168-transrepform.docx]

***eLife’s* transparent reporting form**

We encourage authors to provide detailed information *within their submission* to facilitate the interpretation and replication of experiments. Authors can upload supporting documentation to indicate the use of appropriate reporting guidelines for health-related research (see [EQUATOR Network](http://www.equator-network.org/%20)), life science research (see the [BioSharing Information Resource](https://biosharing.org/)), or the [ARRIVE guidelines](http://www.plosbiology.org/article/info:doi/10.1371/journal.pbio.1000412) for reporting work involving animal research. Where applicable, authors should refer to any relevant reporting standards documents in this form.

If you have any questions, please consult our Journal Policies and/or contact us: [editorial@elifesciences.org](mailto:editorial@elifesciences.org).

**Sample-size estimation**

- You should state whether an appropriate sample size was computed when the study was being designed
- You should state the statistical method of sample size computation and any required assumptions
- If no explicit power analysis was used, you should describe how you decided what sample (replicate) size (number) to use

Please outline where this information can be found within the submission (e.g., sections or figure legends), or explain why this information doesn’t apply to your submission:

Integrative modeling ensemble statistics is described in Figure S4 and Supplementary Computational Methods. Modeling scripts and final models and localization densities are available at https://salilab.org/gtuscSpc110 and have been deposited to the Protein Data Bank archive for integrative structures (https://pdb-dev.wwpdb.org/) with depositions codes PDBDEV_00000077 PDBDEV_00000078 PDBDEV_00000079.

**Replicates**

- You should report how often each experiment was performed
- You should include a definition of biological versus technical replication
- The data obtained should be provided and sufficient information should be provided to indicate the number of independent biological and/or technical replicates
- If you encountered any outliers, you should describe how these were handled
- Criteria for exclusion/inclusion of data should be clearly stated
- High-throughput sequence data should be uploaded before submission, with a private link for reviewers provided (these are available from both GEO and ArrayExpress)

Please outline where this information can be found within the submission (e.g., sections or figure legends), or explain why this information doesn’t apply to your submission:

Cryo-EM data collection and processing statistics can be found in Table S2. A description of cryo-EM data analysis, and classification methods can be found in the Methods section, and in figures S5, S6 and S13.

XL-MS experiments and data collection are described in the Methods section. All raw and processed data, along with the processing software versions and configuration files required to repeat the analyses performed here, can be found at <https://proxl.yeastrc.org/proxl/p/cm1-tusc> as described in the Methods section.

Integrative modeling ensemble statistics is described in Figure S4 and Supplementary Computational Methods. Modeling scripts and final models and localization densities are available at https://salilab.org/gtuscSpc110 and have been deposited to the Protein Data Bank archive for integrative structures (https://pdb-dev.wwpdb.org/) with depositions codes PDBDEV_00000077 PDBDEV_00000078 PDBDEV_00000079.

**Statistical reporting**

- Statistical analysis methods should be described and justified
- Raw data should be presented in figures whenever informative to do so (typically when N per group is less than 10)
- For each experiment, you should identify the statistical tests used, exact values of N, definitions of center, methods of multiple test correction, and dispersion and precision measures (e.g., mean, median, SD, SEM, confidence intervals; and, for the major substantive results, a measure of effect size (e.g., Pearson's r, Cohen's d)
- Report exact p-values wherever possible alongside the summary statistics and 95% confidence intervals. These should be reported for all key questions and not only when the p-value is less than 0.05.

Please outline where this information can be found within the submission (e.g., sections or figure legends), or explain why this information doesn’t apply to your submission:

Processing and statistical information on X-ray experiments is presented in Table S1, and described in Methods. Statistical information on cryo-EM experiments is presented in Table S2, as well as Figures S5, S6 and S13, while methods are described in the Methods section. Sequences and methods used for the visualization of conservation shown in Figure S11 are described in the Methods section.

XL-MS processing statistics are described in the Methods section. Additional detail can be found in the articles referenced in that section. Statistical software versions, commands used, input and output files are described in the Methods section, the references therein as well as <https://proxl.yeastrc.org/proxl/p/cm1-tusc>.

Integrative modeling ensemble statistics is described in Figure S4 and Supplementary Computational Methods. Modeling scripts and final models and localization densities are available at https://salilab.org/gtuscSpc110 and have been deposited to the Protein Data Bank archive for integrative structures (https://pdb-dev.wwpdb.org/) with depositions codes PDBDEV_00000077 PDBDEV_00000078 PDBDEV_00000079.

(For large datasets, or papers with a very large number of statistical tests, you may upload a single table file with tests, Ns, etc., with reference to sections in the manuscript.)

**Group allocation**

- Indicate how samples were allocated into experimental groups (in the case of clinical studies, please specify allocation to treatment method); if randomization was used, please also state if restricted randomization was applied
- Indicate if masking was used during group allocation, data collection and/or data analysis

A description of cryo-EM data analysis, and classification methods can be found in the Methods section, and in figures S5, S6 and S13.

Please outline where this information can be found within the submission (e.g., sections or figure legends), or explain why this information doesn’t apply to your submission:

**Additional data files (“source data”)**

- We encourage you to upload relevant additional data files, such as numerical data that are represented as a graph in a figure, or as a summary table
- Where provided, these should be in the most useful format, and they can be uploaded as “Source data” files linked to a main figure or table
- Include model definition files including the full list of parameters used
- Include code used for data analysis (e.g., R, MatLab)
- Avoid stating that data files are “available upon request”

Please indicate the figures or tables for which source data files have been provided:

Structure factors and model coordinates for the Xrcc4-Spc110p^164-207^ fusion X-ray crystal structure have been uploaded to the RCSB protein data bank with PDB ID 7M3P.

Cryo-EM reconstructions and model coordinates have been deposited to the EMDB and PDB for the γTuSC monomer (EMDB ID: EMD-23638 PDB ID: 7M2Z), γTuRC^SS^ (EMDB ID: EMD-23635 PDB ID: 7M2W), γTuRC^WT^ open (EMDB ID: EMD-23636 PDB ID: 7M2X) and γTuRC^WT^ closed (EMDB ID: EMD-23637 PDB ID: 7M2Y) states. The cryo-EM reconstruction for the γTuSC dimer (EMDB ID: EMD-23639) has been deposited to the EMDB. Accession codes are also available in Tables S1 and S2.

XL-MS experiments and data analysis are described in the Methods section. All raw and processed data, along with complete information required to repeat the current analyses, can be found at <https://proxl.yeastrc.org/proxl/p/cm1-tusc> as described in the Methods section. In addition, the complete crosslinking dataset and analysis presented in this paper can be viewed, downloaded, examined and visualized using our web-based interface, ProXL, at the URL above.

Integrative modeling scripts and final models and densities are available at https://salilab.org/gtuscSpc110 and have been deposited to the Protein Data Bank archive for integrative structures (https://pdb-dev.wwpdb.org/) with depositions codes PDBDEV_00000077 PDBDEV_00000078 PDBDEV_00000079.
